# Lipid-Based Nanoparticles as Oral Drug Delivery Systems: Overcoming Poor Gastrointestinal Absorption and Enhancing Bioavailability of Peptide and Protein Therapeutics

**DOI:** 10.34172/apb.2024.016

**Published:** 2023-10-14

**Authors:** Soheil Mehrdadi

**Affiliations:** Department of Pharmaceutical and Pharmacological Sciences, University of Padova, Padua, Italy.

**Keywords:** Peptide and protein therapeutics, Lipid-based drug delivery systems, Solid lipid nanoparticles, Nanostructured lipid carriers, Oral drug delivery

## Abstract

Delivery and formulation of oral peptide and protein therapeutics have always been a challenge for the pharmaceutical industry. The oral bioavailability of peptide and protein therapeutics mainly relies on their gastrointestinal solubility and permeability which are affected by their poor membrane penetration, high molecular weight and proteolytic (chemical and enzymatic) degradation resulting in limited delivery and therapeutic efficacy. The present review article highlights the challenges and limitations of oral delivery of peptide and protein therapeutics focusing on the application, potential and importance of solid lipid nanoparticles (SLNs) and nanostructured lipid carriers (NLCs) as lipid-based drug delivery systems (LBDDSs) and their advantages and drawbacks. LBDDSs, due to their lipid-based matrix can encapsulate both lipophilic and hydrophilic drugs, and by reducing the first-pass effect and avoiding proteolytic degradation offer improved drug stability, dissolution rate, absorption, bioavailability and controlled drug release. Furthermore, their small size, high surface area and surface modification increase their mucosal adhesion, tissue-targeted distribution, physiological function and half-life. Properties such as simple preparation, high-scale manufacturing, biodegradability, biocompatibility, prolonged half-life, lower toxicity, lower adverse effects, lipid-based structure, higher drug encapsulation rate and various drug release profile compared to other similar carrier systems makes LBDDSs a promising drug delivery system (DDS). Nevertheless, undesired physicochemical features of peptide and protein drug development and discovery such as plasma stability, membrane permeability and circulation half-life remain a serious challenge which should be addressed in future.

## Introduction

 In recent decades, high demands for cost-benefit healthcare expenses, efficient therapeutics (e.g. safety and efficacy) and non-stop generic substitution has urged pharmaceutical companies,^1^ industry and market to shift to biotechnology-driven peptide and protein therapeutics and biopharmaceuticals. These novel categories of drugs, unlike active pharmaceutical ingredients (APIs, drugs), offer better feedback due to higher potency, selectivity and specificity for their extracellular target.^2^

**Figure 1:**
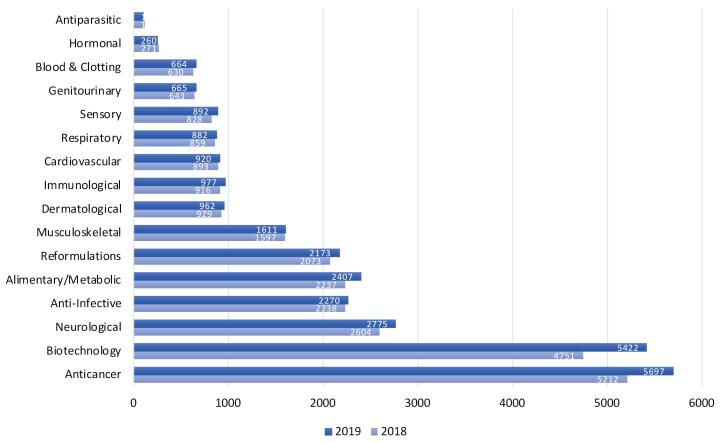
Biotechnology medicines in research and development (R&D) by therapeutic category

 Peptides and proteins, as cell products, have various physiological functions in body such as hormones, enzyme substrates and inhibitors, antibiotics, biological regulators, structural components, signaling factors and catalyzers which all implies their importance in body; hence, any abnormality in their amino acid sequence or structural disfunction leads to sever diseases and pathological conditions; diabetes,^3^ dwarfism,^4^ cystic fibrosis,^5^ thalassemia^6^ or impaired blood clotting,^7^ among many others.^8,9^ as such, due to their biological specificity and efficient affinity and efficacy, peptides and proteins have been exploited as drugs for treatment of diseases (Figure 1).^10^

 Stability in proteins is the result of balance among four destabilizing and stabilizing forces: electrostatic interactions, hydrogen bonding, van der Waals forces and hydrophobic interactions, which form the secondary, tertiary and quaternary structures of proteins and any disruption will influence the structural balance and destabilizes that particular protein.^11,12^ Different environmental factors can influence the chemical and physical stability of proteins such as pH, ionic strength, temperature, high pressure, non-aqueous solvents, metal ions, detergents, adsorption, agitation and shearing which all are inevitably part of the manufacturing, sterilization and lyophilization process, and consequently might damage the developing protein resulting in biological inactivation, aggregation, immunogenicity and precipitation.^13-15^

 Peptide and protein therapeutics are highly dependent on the production process, yet are biocompatible, cost-benefit, with modifiable in-vivo bioactivity, specific targeting, chemical diversity, and easily synthesized by using solid-phase peptide synthesis methodologies (e.g. Merrifield’s method) in which the amino acid sequence can be precisely chosen and inserted at the molecular level by modifying the basic units.^16^ nevertheless, undesired physicochemical features of protein drug development and discovery, remains a serious challenge for formulation scientists as well as pharmaceutical and biotechnology companies.

 Only recently peptides have been considered as therapeutic agents while they were never considered as a potential therapeutic agent^17^; mostly due to their protease degradation, metabolic instability, short half-life, manufacturing complications and high expenses, which in long-term administration renders them unfavorable in terms of patient costs and compliance especially with regard to parenteral administration as the majority of peptides (10%) have a very low oral bioavailability.^18^ On the other hand, peptides’ biodegradability into non- to low-toxicity metabolites,^2^ low drug-drug interactions and immunogenicity,^2^ higher tissue penetration (owing to their small size), higher in-vivo activity (per unit mass), stability and lower expenses favors them over large therapeutic proteins and antibodies for regulatory approval (higher than 20%) which is twice the rate of small molecules.^17^

 As well as peptides, proteins also gained importance and their application in pharmaceutical science and industry was emphasized due to more advanced analytical methods resulting in the recognition of various peptides and hormones as therapeutic biopharmaceuticals, novel genetic and molecular engineering methods to produce large-scale proteins and recently-defined roles of proteins as regulatory components of numerous diseases.^19,20^ Since then, pharmaceutical industry has developed various large-scale oral delivery technologies for peptides/proteins as active ingredients.^21^

 Low oral bioavailability of peptide and protein therapeutics renders them being formulated as parenteral preparations; large molecular size,^22^ susceptibility to enzymatic degradation (local or mostly GI tract), poor stability in the gastric acidic environment,^23^ poor intestinal penetration, short plasma half-life, immunogenicity and the propensity to aggregation, adsorption, and denaturation.^24,25^ Enzymatic degradation and poor intestinal penetration, among all, have been mainly mentioned for low oral bioavailability and short half-life of protein drugs ( < 1%) which is claimed to be increased to 30%-50% by pharmaceutical enterprises.^26,27^ Pharmaceutical therapeutic proteins, due to their large molecular size which leads to low blood absorption and diffusion, requires specific epithelial transporters otherwise they cannot enter the general circulation by the ordinary routes of drugs ansorption. Furthermore, low pH and protease enzymes of GI aggravate this condition even more.^28^

 Among different administration routes, the most common route for peptide and protein therapeutics is intravenous (I.V.) injections (Table 1), which is not favorable in terms of patient compliance, clearance varies from a few minutes to several days, and might result in undesired deposition and distribution which require repeated injections with higher therapeutic doses to achieve efficacy,^29,30^ subsequently causing severe adverse effects.

**Table 1:** Various peptides and proteins’ administration routes

**Method**	**Delivery routes**	**Formulation/Device requirement**	**Reference**
Invasive	Intravenous (I.V.), subcutaneous (S.C.), intramuscular (I.M.), Intracerebral vein (I.C.V.)	Liquid or reconstituted solid (syringe), i.v. injected liposomes	^31-35^
	Depot system (S.C. or I.M.)	Biodegradable polymers, liposomes, permeable polymers (not degradable) microspheres, implants	^31-33,36,37^
Non-invasive	Pulmonary	Liquid or powder formulations, nebulizers, metered dose inhalers, dry powder inhalers	^31-34,38^
	Oral	Solids, emulsions, microparticles, nanoparticles, with or without absorption enhancers	^31-33,38,39^
	Nasal	Liquid, usually requires permeation enhancers, nanoparticles	^31-33,40,41^
	Transdermal	Iontophoresis, electroporation, chemical permeation enhancers, prodrugs, sonophoresis, transfersomes	^31-33,42,43^
	Buccal, rectal, vaginal, ocular	Gels, suppositories, bioadhesives, microparticles	^31-33,44,45^

 The subcutaneous (S.C.) and intramuscular (I.M.) injections are other administration routes (Table 1), the former is the most common one, especially for vaccines. Different factors such as molecular weight, site of injection, local injection site activity and pathological conditions can influence the fate of peptide and protein therapeutics following S.C. injection leading to a bioavailability as high as 100% or much lower.^46^ upon injection, proteins with high molecular weight ( < 16 000 Da) are absorbed either from vessels’ endothelial cells to capillaries or reach the local lymphatic system and through thoracic duct join the blood circulation, while proteins with small molecular weight are mainly diffused through the local capillaries.^46^ however, protein transportation through lymphatic way is undesired due to slow time of circulation which might result in protein enzymatic degradation.^46,47^

 Non-invasive routes have increasingly been investigated as well as an alternative to the conventional invasive injectable routes (Table 1). Numerous studies have investigated nasal, ophthalmic, buccal, rectal, vaginal, transdermal and pulmonary routes for peptide and protein delivery.^28,48-55^

 Based on studies, mucosae which so far have been neglected for drug delivery seem to be a promising approach for drug absorption, especially efficient for biomolecules of large size and molecular weight.^28,56^ The advantages of mucosal surfaces (mouth, eye, nose, rectum and vagina) for drug delivery over skin and GI tract can be named as: fewer biological barriers to pass for systemic diffusion, rapid absorption and evading hepatic first-pass effect. However, one practical challenge of mucosae is related to the preparations that are formulated for local long-term treatment.

 Despite alternate routes of drug delivery, oral delivery (P.O.) is still the most preferred one; non-invasive, painlessness, easy self-administration, low risk of cross-infection, high patient convenience/compliance, outpatient feasibility,^57^ cost-benefit (no need for sterile manufacturing).^57^ Oral route does not offer the drawbacks of I.V. route; drug extravasation from blood, catheter-related infectious complications, thrombosis and being expensive and invasive, especially for chronic conditions.

 However, biological barriers of GI tract with their associated enzymatic and chemical processes hamper the efficiency of oral route for drug delivery. Furthermore, the epithelial cell monolayer membrane of the GI tract even more aggravate the condition of low permeability for many peptide and protein therapeutics with low gastrointestinal solubility which finally results in low bioavailability.^58^

 Some active moieties cannot be delivered through oral route.^59,60^ According to the Biopharmaceutic Classification System (BCS),^61^ oral bioavailability of each drug is determined by its solubility along the GI tract and cellular penetration. Most of the potential drug candidates developed with high-throughput screening methods generally have higher molecular weights and tend to be lipophilic in nature.^62^ Other factors contributing to low oral bioavailability of drugs are low stability in the gastrointestinal environment and poor membrane permeability. Most of drugs are substrates to intestinal efflux transporters like p-glycoprotein resulting in poor oral bioavailability.^63^

 Regardless of the administration route, majority of peptide and protein therapeutics, lacking necessary physicochemical requirements, fail to diffuse and be absorbed to their target tissue which implies the importance of drug delivery and tissue-targeting systems for achieving as maximum therapeutic effects as possible. The carrier systems diffuse and distribute the intended therapeutic molecules to their targeted site with as maximum concentration as possible in the affected area and as minimum concentration as possible in the intact tissues to lower the general adverse effects.^64^

## Peptide and Protein Drug Delivery

 Introduction of novel biotechnological molecules as potential therapeutics, advent of chemical synthesis methods and recombinant DNA technology have all rendered protein synthesis and delivery an important area of research which resulted in the production of numerous large-scales drugs of peptide/protein origin such as monoclonal antibodies, hormones and vaccines. According to The 2018 and 2019 PhRMA reports,^65^ there have been respectively 4751 and 5422 novel biotechnological medicines in research and development (R&D) phase for more than 100 diseases such as cancer, infectious diseases, autoimmune diseases, AIDS/HIV, antiparasitic and related conditions (Figure 1), which have been either in human clinical trials or under review by the Food and Drug Administration (FDA).^65^ nevertheless, abovementioned challenges for their delivery through GI tract ^66-70^ and the blood brain barrier (BBB)(in the case of central nervous system diseases),^71,72^ makes their therapeutic potential and clinical application questionable.

 In the last decades, numerous drugs of peptide/protein origin have been in preclinical studies and clinical trials,^73^ more than 400 recombinant peptides and proteins and 1300 under clinical trials.^74^ The reason could be attributed mostly to the larger size of peptides and proteins comparing to conventional drugs, which provides drug-target interaction with binding pockets that are not normally available to small molecular drugs. These targets could be part of intracellular protein-protein interaction network which have been recognized in numerous diseases. Peptide and protein therapeutics in order to interact with such targets must penetrate cells, however, most of them are known to have extracellular targets,^73^ and are parenterally administered so cellular penetration is not their ordinary route as it is for mucosal surfaces. Currently, the main obstacle of the oral administration of these novel categories of drugs for their maximum therapeutic effects could be addressed as the penetration through intestinal cellular membranes and target cellular membranes.

## Transport mechanisms in the GI tract

 To formulate and synthesize drug delivery systems (DDSs) for oral peptide and protein therapeutics, and biopharmaceuticals a throughput understanding of the biological pathways involved for their absorption and diffusion in the GI tract is necessary and worthwhile. Various physicochemical features govern the pathway through which the molecules will be penetrating the intestinal cells; molecular weight, hydrophobicity/hydrophilicity, ionization constants, and pH stability, among all.

###  Paracellular transport

 It has the following features as, space dimension of 10 Å, aqueous pores (epithelial tight junctions) 7–9, 3–4 and 8–9 Å for the jejunum, the ileum and the colon respectively,^75^ to allow the passage of solutes with a specific molecular radius and tight junctions building 0.01% of the total absorption surface area of the intestine.^76^ These data prove the restriction of the paracellular transport toward the passing molecules (Table 2). however, there is an electrical resistance diversity and consequently ionic selectivity. In the latter case, also transcellular pathway’s collaboration adjusts rate and selectivity of export of ions and solutes and overall tissue-specific transport. The tight junction along with ion channels are involved in size and charge selectivity, ion concentration-dependent penetration, competitive-based penetration among different molecules, unordinary mole-fraction effects and pH-sensitivity.^77^ hydrogen bonding capacity and lipophilicity do not influence much the paracellular pathway.

**Table 2:** Transport mechanisms in the GI tract

**Transport Mechanisms**	**Process**	**Feature of Molecules**	**Note**
Paracellular	Passive diffusion in intercellular spaces between epithelial cells, tissue-specific transport^78,79^	Ions, large substances and solutes < 15 Å (3.5 kDa)^80^	Restricted protein delivery due to tight junctions^81^
Transcellular	Intestinal transcytosis, Enterocytes and M cells^82^	Various physicochemical properties	Limited transport of relatively low molecular-weight lipophilic drugs
Carrier-mediated	Across the cell membrane or entire cell^83^	Utilized by small hydrophilic molecules ^84^	Small di-/tripeptides monosaccharides, and amino acids are transported transcellularly^85^
Receptor-mediated	Receptor specific ligand,^86^ endocytosis^87^	The physicochemical and metabolic features	Direct delivery of hydrophilic ligands to liver, direct delivery of lipophilic ligands to the vena cava^88^

###  Transcellular transport

 It is an endocytic process at apical membrane and the absorbed molecules are released at the basolateral membrane, glucose is also transported with this mechanism (Table 2). The protein-lipid ratio is very insignificant in the basolateral membrane due to its thinner and more permeable structure than the apical membrane. This transport mechanism is governed by various factors: different physicochemical properties of molecules, size, lipophilicity/hydrophobicity, hydrogen bond formation, surface charge, superficial ligands; the physiological condition of the GI system and the animal models studied for transport mechanism.^89,90^ There are mainly two types of primary intestinal epithelial cells for molecules transportation; Enterocytes and M cells, the former lining about 99% of the GI tract and the latter mainly the area of Peyer’s patches and the human follicle-associated epithelium (FAE) (antigen-specialized).^91^ M cells function as presenting and transporting peptides and proteins to the local lymphoid tissues for immune reactions and a vulnerable and available way for pathogenic organisms.^92^ Due to their great endocytosis and transcytosis capacity for transporting diverse molecules and biomaterials (e.g. nanoparticles),^93,94^ M cells could be used for oral delivery of peptides and proteins and finally through phagocytosis, adsorptive (through clathrin-coated pits and vesicles) and fluid phase endocytosis they adsorb macromolecules and microorganisms.^95^ Some studies demonstrated nanoparticles transportation through intestinal villi and contradicting the recent debates over the rate of particle absorption.^96,97^ There is a consensus on the transportation of the majority of particles in FAE,^96,98,99^ for which there have been studies on the Peyer’s patches and M cells involvement on various biomaterials absorbency. The transcellular mechanism however is not a desirable route for low molecular-weight lipophilic drugs. Overall, absorption by this mechanism is reduced in a great extent in the colon part of the large intestines in comparison to the paracellular mechanisms.^100^

###  Carrier-mediated transport

 It is an active and energy-dependent transportation of specific molecules against their concentration gradient through specific membrane receptors, such as β-lactam antibiotics and angiotensin converting enzyme inhibitors, monosaccharides and amino acids (Table 2). In one study using Caco-2 cell monolayers, it was proved that the conjugated insulin is transported 5 to 15 times more through the transferrin receptor than then insulin receptor itself.^101^

###  Receptor-mediated transport

 It has been investigated to evaluate the oral bioavailability of peptide and protein drugs by modifying receptor specific ligands-drug interaction. This mechanism has functions in different processes such as endocytosis (clathrin-mediated), phagocytosis, pinocytosis and potocytosis (nonclathrin-mediated) (Table 2). The absorption starts with the binding of molecules to their specific receptors and their internalization into endosomes with low acidic pH which might dissociate receptor-ligand bound and accordingly degrades endosomes. The absorbed peptide and protein access into systemic blood circulation with two distinct pathways: hepatic portal vein and intestinal lymphatic vessels, the amount of peptides and proteins absorbed through either of these two pathways depends greatly on the physicochemical features of the formulation. portal vein is the main pathway for the majority of orally administered peptide and protein drugs and through which hydrophilic molecules are absorbed and transported to the blood systemic circulation first through the hepatic portal vein and then by the hepatic artery, and finally they are delivered to their sites of action. But lipophilic molecules penetrating through the same intestinal barriers are transported to the intestinal lymphatic vessels, bypassing the first-pass effect, and directly delivered to the vena cava for blood systemic circulation.

## Absorption of oral drugs

 Drugs in order to be absorbed through GI tract are required to have high solubility and permeability, however, this is not the case for numerous drugs with low aqueous solubility and consequently low and diverse bioavailability.^102^ for such drugs simultaneous presence of high amount of fat through meals can increase their oral bioavailability,^103-105^ via prolonging GI tract passage time, exocrine pancreas secretion stimulation, reduced metabolism, lymphatic-associated absorption, increased intestinal penetration, reduced cellular efflux and liver- and mesenteric-related blood alteration.^106,107^

 Introduction of lipid-based drug delivery systems (LBDDSs) in 1990s provided scaffolds which increase dissolution rate of poor aqueous soluble drugs (i.e. hydrophobic drugs) by providing a phase in which the drugs can disintegrate and be absorbed and diffused toward its site of action.^108^ after the degradation of lipids in the intestine, active mono- and diglycerides are formed on the surface of lipids which later disassociate and transform into micelles and simultaneously drug is also solubilized inside micelles. These micelle-drug mixtures are finally absorbed.^109-112^

 The absorbed components through intestinal segment of GI system follow two distinct pathways according to their features: blood vessels and lymphatic vessels. The former is the preferred route for most of the oral drugs by which they are absorbed into the systemic circulation via portal vain, and the latter for highly lipophilic drugs (log *P* > 5) by which drugs are absorbed into systemic circulation via lymphatic vessels.

 Overall, the presence of lipid increases absorption of numerous drugs more through lymphatic vessels, especially lipophilic ones and macromolecules of high molecular weight,^113,114^ mostly due to their higher permeability to nanoparticles than blood vessels,^115^ and most importantly overpassing hepatic first-pass effect.^116^ Nevertheless, it has been demonstrated that the absorption through lymphatic pathway is affected by the length of fatty acid chains; long-chain triglycerides (14-18 chains) are more favored for absorption than low-chain ones.^111,117^

## Physiological barriers against peptide and protein therapeutics absorption

###  Gastrointestinal barriers

 In-depth studies of molecular pharmacology have provided a better understanding of the biological and molecular processes of the GI system, while its target sites has introduced new insights for targeted delivery of oral peptide and protein therapeutics and biopaharmaceuticals.^118,119^ There are two types of proteolytic enzymes with their specific site of action which are responsible partly for the physiological processes of GI system toward peptides and proteins: *endoproteases*, including trypsin, chymotrypsin, and elastase, which hydrolyze the internal bonds of the peptide chain to the amino- and carboxy-terminus, and *exopeptidases*, including carboxypeptidase A and aminopeptidase, which hydrolyze the amino- and carboxy-terminus bonds to the peptide chain (Table 3).^120^ Enzymatic degradation happens at the lumen, brush border, the cytosol of the enterocytes, in the lysosomes and other cell organelles.^121^

**Table 3:** Proteases and their sites of action

**Types **	**Enzymes**	**Major Site of Action**
Gastric proteases	Pepsins (aspartic proteases)	Broad activity, hydrolyzes numerous peptide bonds
Brush border proteases	Aminopeptidase A Aminopeptidase N Aminooligopeptidase Dipeptidylaminopeptidase IV Carboxypeptidase	Aminopeptidases are N-terminopeptidases, degrading mostly 3–10 amino acid residue-dipeptides and amino acids
Cytosolic proteases	Di- and tripeptidase	2-3 Aminopeptide amino acids
Intestinal pancreatic proteases	Trypsin (endopeptidase) α-chymotrypsin (endopeptidase) Elastase (endopeptidase) Carboxypeptidases (exopeptidase)	Peptide bonds of basic amino acids/peptides Peptide bonds of hydrophobic amino acids/peptides Peptide bonds of smaller and nonaromatic amino acids/peptides A: C-terminal amino acid B: C-terminal basic amino acid
Brush border proteases	Aminopeptidase A Aminopeptidase N Amino oligopeptidase Dipeptidyl aminopeptidase IV Carboxypeptidase	Aminopeptidases are N-terminopeptidases, degrading mostly 3–10 amino acid residue-dipeptides and amino acids

 The secretions of stomach (hydrochloric acid, potassium chloride and sodium chloride) provide an acidic pH of 1.5-3.5 for the proteolysis of peptides and proteins by breaking them down into amino acids, dipeptides and tripeptides for absorption. The digestion of peptides and proteins starts with pepsin in the acidic environment of stomach (pH 2), however, the alkaline environment of the intestines (pH 6) inactivates pepsin. The instantaneous wide change of pH from acidic stomach to alkaline intestines influences the degradation of ingested peptides and proteins and might contribute to their precipitation that redissolve following pH change.^122-124^

 The small intestine is the major site of absorption along the GI tract due to the higher enzymatic activity of proteases (mostly in duodenum and jejunum). The brush borders of the intestinal epithelial cells secrete numerous specific enzymes (e.g. sucrose) leading to peptide/protein absorption and degradation.^120^ furthermore, the digestive secretions of the exocrine part of the pancreas also contain endo-/exopeptidases which are released into duodenum to increase pH for the activity of intestinal enzymes (e.g. trypsin). However, in the terminal parts of jejunum and ileum the enzymatic activity of aminopeptidases is decreased to 20–30% where Peyer’s patches are located and these areas could be a potential site for peptide/protein drug delivery.^98,125^

###  Mucosal barriers

 Mucus has a major importance and function by determining the absorption and bioavailability of administered drugs especially through oral route. The mucosal surface of stomach has three components which further hamper the drug diffusion and absorption as an exogenous component: the first one, which is lined by surface epithelial cells and tight junctions, has a role against irritant and unsuitable fluids; the second one has a very unique insoluble protective mucus (the mixture of surface epithelial cells and neck cell) which creates a jelly-like layer throughout the entire surface mucosae of the stomach; and the third one is composed of bicarbonate ions which are secreted by the surface epithelial cells.^126,127^

 There are however other components which further disturb peptide/protein absorption through oral administration, such as glycocalyx located on the surface layer of the stomach epithelial cells with an acidic nature and containing sulfated mucopolysaccharides. Goblet cells of the stomach wall secrete mucus, which covers upper layer of glycocalyx,^128^ and contains mucin glycoproteins, enzymes, electrolytes and water.^129^ the mucin glycoprotein gives glycocalyx an adhesive feature and functions more as a physical barrier than a chemical one,^130,131^ and in the stomach and colon parts of GI tract has a thick layer while in the small intestine part is thinner and this fact can be justified according to the digestive functions of each segment.^132^ Overall, the abovementioned components altogether are protected by viscoelastic layers. Peptide and proteins first of all are required to pass the outermost layers, mucus and glycocalyx, to reach the cellular membranes which present a viscous barrier to absorption and diffusion.

## Nanomedicines – novel drug delivery systems

 After administrating drugs, their plasma concentration increases and they affect their site of action effectively followed by a gradual decrease to a point when their plasma concentration is not sufficient enough anymore to elicit their intended therapeutic effect. Hence, Drugs are required to be re-administrated based on the dose and frequency of administration to provide the same concentration which must be neither higher nor lower than their therapeutic concentration level; a concept widely known as “therapeutic window”; higher doses will result in general toxic effects and lower ones cannot elicit any therapeutic effects.^133^ the conventional drugs do not have prolonged drug delivery features in their “therapeutic window” and this issue implies the importance of novel DDSs to be introduced.

 A novel interdisciplinary branch of biomedical science, “nanomedicine”, has been investigating the potential application of biomaterials and nanostructures as DDSs for sustained, controlled and tissue-targeting delivery of drugs and active agents, which due to their pharmaceutical features overcome some of the limitations of conventional drugs.

 Studies have proved that “drug discovery” alone does not offer practical solutions to therapeutics as most of the successful in-vitro experiments result in failure in in-vivo experiments mostly due to: poor drug concentration owing to low absorption, quick metabolism and elimination (peptides and proteins); general blood distribution which results in drug-related adverse effects and toxicity (anti-cancer drugs); low drug solubility of aqueous solutions when administered intravenously; unpredictable bioavailability and high plasma-level variations with oral administration and physiological processes involving a drug’s plasma level (e.g. food on cyclosporine).

 In DDSs the in-vivo destiny of drugs is directly influenced by a series of factors which can be modified for in-site delivery with the desired therapeutic concentration. Peptide and protein therapeutics are encapsulated in nanomedicines for transportation through GI tract offering benefits such as high stability for storage and administration, and large-scale sterile manufacturing for oral preparations.^134^ Nanomedicines can be formulated and modified with the desired criteria such as size, surface properties and release profile to have tissue-targeted delivery within drug’s unique therapeutic window.

 Nanomedicines have a size ranging from 1 to 100 nm where the therapeutic molecules can be incorporated in the core, matrix or attached on the surface (in the case of high surface/volume ratio),^135^ which the latter results in longer half-life and systemic circulation and increased mean residence time (MRT).^136^ Since 1990s LBDDSs have been under investigation owing to their advantages; biocompatibility, higher penetration capacity, lipophilicity with no need for surface modification, simple fabrication, cost benefit and industrial-scale production compared to their counterpart DDSs such as polymeric and inorganic nanomedicines.^137,138^

 Based on the fabrication methods and physicochemical properties LBDDSs are classified into the following:

 Liposomes in the form of spherical vesicles which are composed of one or multiple lipid bilayer (phospholipid or natural phospholipids) enclosing an aqueous core.^137,138^ Their size varies from 10 to 1000 nm and they are the first generation of LBDDSs that were employed mainly for parenteral route of drug delivery. They possess low antigenicity and toxicity, high drug encapsulation and loading efficiency and sustained and controlled drug release as their benefits. However, their synthesis is complex and has low stability and rapid reticuloendothelial system clearance and scale-up problems. Despite introducing various surface functionalization (antibodies and peptides) and modification (e.g. PEG coating) which enhances their blood half-time, structural stability and therapeutic efficiency, they are still undesirable in terms of industrial scale production.^139-141^ Currently, numerous liposome-based formulations are approved for a variety of diseases: Doxorubicin for cancers (Doxil®, Myocet® and Lipodox®), Amphotericin B for fungal infections (Ambisome®), Cytarabine for lymphomatous meningitis (Depocyt®), Morphine sulfate for pain management (DepoDur®), inactivated hepatitis A virus (strain RG-SB) for hepatitis A (Epaxal®) and inactivated hemagglutinin of Influenza virus strains A and B for influenza (Inflexal®).^142^

 Lipoplexes are fabricated from liposomes and are designed in multi-lamellar lipoplexes with positive lipid bilayer and distinct negative nucleic acids. The electrostatic process between self-assembly liposomes and nucleic acids produces such scaffolds. Given their similarity to liposomes they possess similar benefits and drawbacks with a few of their own; multiple cations tendency to bind negative nucleic acids which decreases the internal cell transfection process. They have been investigated for brain-focused studies.^143^

 Natural body lipoproteins are another lipid-based system which are very similar to liposomes hence sharing the same advantages and disadvantages and carry lipids (mainly cholesterol), proteins, enzymes, and miRNAs. They have been investigated with other nanoparticles (e.g., albumin, PEG-PLGA) for numerous central nervous system diseases.^144^

 (2) Niosomes are vesicles with a lamellar self-assembled structure which are composed of non-ionic surfactants and cholesterol or its derivatives.^145^ They can be encapsulated by lipophilic and hydrophilic substances. They are cheaper in terms of production and more stable than liposomes.^146^

 (3) Transferosomes which are composed of a lipid bilayer fabricated by a lipid matrix stabilized by various surfactants,^147^ and are similar to niosomes and to liposomes.

 (4) Solid lipid nanoparticles (SLNs) which are composed of a solid lipid core.^148^

 (5) Nanostructured lipid carriers (NLCs) which are composed of a liquid lipid phase core within the solid lipid phase.^149^

 Among all the lipid-based DDSs, this review will focus further on SLNs and NLCs and their unique characteristics and properties. SLNs, comparing to NLCs which are formulated with both solid and liquid oils, are formulated only with solid ones which gives them more controlled drug release due to limited drug mobility in solid lipids and are designed as oral pellets and retard capsule (e.g. Mucosolvan®), as microparticles by spray drying,^150^ and oral nanopellets.^151^

## Solid lipid nanoparticles and nanostructured lipid carriers

 SLNs and NLCs are lipid-based colloidal drug carriers, synthesized from lipids (solid or liquid), surfactants, co-surfactants and APIs (drugs). SLNs are composed of solid lipids and surfactants but NLCs are also composed of liquid lipids and oils. Lipids have solid form both at room and body temperature and could be chosen as purified triglycerides, glyceride mixtures and waxes. Surface surfactants increase and improve the stability and cellular permeability and consequently absorption.^152,153^

 They collectively offer the benefits of other colloidal DDSs (e.g. liposomes and polymeric NPs) and avoid their drawbacks;^154^ enhanced dissolution rate, bioavailability, tissue distribution, encapsulation rate, absorption, stability of drug in body fluids, no unpleasant taste (exists with oral preparations), lower toxicity, no organic solvents usage, large-scale production possibility, sterility, reduced first-pass effect and controlled and sustained tissue-targeted delivery with various routes of administration: oral, parenteral, nasal, rectal, ophthalmic, etc.^155^

 Due to some drawbacks of SLNs such as low drug loading capacity, unpredictable gelation tendency, and drug expulsion after polymorphic transition during storage,^152-156^ NLCs were introduced and synthesized as the improved version of lipid-based nanocarriers with numerous methods exploited to formulate and prepare them.^155-157^ Hence, the final nanoparticle characterization must comply with the dynamic processes and such a characterization is the real challenge to represent the highest quality for the product,^157^ storage drug expulsion, unpredictable gelation and their co-encapsulation with nano- and micro-sized structures owing to high lipid concentration and surfactants might influence the in-vivo fate of nanoparticles.

 The major criteria for the characterization of nanoparticles as efficient and safe DDSs could be addressed as: size, encapsulation efficiency, structure, co-existence of material, surface morphology and functionalization, and minimum drug dose, which arguably influence directly the bioavailability, absorption and distribution of the encapsulated drug (Table 4).

**Table 4:** Models of drug incorporation for the lipid nanoparticles

**Model**	**Drug Loading Site**	**Drug Release Pattern**
Homogenous matrix of solid solution^158,159^	Homogeneous drug dispersion in the lipid matrix of the particles	Diffusion from the solid lipid matrix and/or by degradation of lipid matrix in GI
Drug-enriched shell^158,159^	Drug concentration on the outer shell of the nanoparticles	Burst release^160^ modified by varying the formulation conditions: production temperature (preferably cold homogenization) and surfactant concentration^161^
Drug-enriched core^158,159^	Drug concentration in the core of the nanoparticles	Prolonged drug release^161^

## Types of solid lipid nanoparticles

 Type 1: Drug molecules/APIs are dispersed either in the lipid core or as amorphous clusters in “the homogenous matrix model”, which offers controlled release features. In order to design and fabricate this type, appropriate concentration of API/lipid ratio must be adjusted using either above-melting point of the lipid or cold methods of high-pressure homogenization (HPH).^162^

 Type 2: This type, known as “Drug enriched shell model”, is designed and fabricated with low concentration of API in the melted lipid. Using the hot method of HPH, the lipid phase is precipitated during the cooling phase which leaves a higher concentration of API in the residue of melted lipid leading to the formation of a free-API lipid core being surrounded by an outer shell composed of the saturated API and lipid. This type does not function for sustained release, however, it does for burst release of API.^162^

 Type 3: Hence “Drug enriched core model”, it is designed by solubilizing the drug in the melted lipid up to its saturation solubility. Following cooling of the lipid the drug is super-saturated in the melted lipid and recrystallizes before the lipid does. Further cooling process renders also lipid recrystallization surrounding the prior formed drug-enriched core. This type offers sustained and prolonged drug release.^162^

## Types of nanostructured lipid carriers

 Imperfect. They are named “imperfect” due to the tiny pores in the solid matrix core which are loaded with API. They are designed by adding and blending solid and liquid lipids (oil) which the co-presence of fatty acids with different chain length (mono-, di- and triacylglycerols) confers an imperfect structure for encapsulating the API.^163^

 Amorphous. They are designed blending lipids which don’t crystallize after homogenization and cooling process,^164^ such as hydroxyl octacosanyl hydroxy stearate, isopropyl myristate and dibutyl adipate, that give them an unorganized amorphous matrix which reduces API repelling of storage and shelf time.^163^

 Multiple. The advantage of the higher solubility of lipophilic drugs in liquid lipids than solid lipids can be used for formulating multiple types of NLC. Solid lipids are mixed with oils which are gradually added in higher amounts exceeding their solubility which results in phase separation of tiny particles with the surrounding solid lipid matrix. This type offers controlled drug release without expelling it out of the lipid matrix.^163^

 Nevertheless, even the lipid-based DDSs have their own advantages and disadvantages (Table 5).

**Table 5:** Advantages and disadvantages of lipid-based drug delivery systems

**Characteristics**	**Biological/Technological aspects**	**Perspectives**	**Reference**
	Various administration routes	Broad-spectrum drug application, therapy optimization	^165^
	Biodegradability	Sustained drug release	^166^
	Controlled drug release	Patients safety, prolonged drug release, in-site drug concentration	^167^
	Site-specific targeting	Decreased systemic toxicity, targeted therapy	^168^
	Biocompatibility	No allergic reactions	^169^
	Increased bioavailability of encapsulated drug	Decreased dose	^170^
	Decreased adverse effects of toxic drugs	Improved patient safety	^171^
	Biological barriers penetration	Various administration routes	^172^
	Reduced dosing frequency	Patients compliance	^167^
Advantages	Chemical and enzymatic degradation drug protection	Possibility of broad-spectrum administration routes	^168^
	Physical stability	Improved drug formulation stability	^173^
	Capacity to encapsulate hydrophilic and hydrophobic drugs	Versatility for different drug groups	^174^
	Scaled up production	Industrial production possibility	^165^
	Simple manufacturing	Easy fabrication in labs, low cost	^166^
	No organic solvents	No toxicity concerns, green chemistry	^169^
	Co-delivery	Offering combined therapy	^166^
	Increased drug loading capacity	Decreased formulation dose	^173^
	Sterilization possibility	Parenteral administration optimization	^175^
	Small size distribution	Potential alternative for drug delivery	^176^
	Initial burst effect of encapsulated drug	Patient overdose risk	^177^
	Low plasma circulation time	Fast reticuloendothelial clearance before in-site deposition	^178^
	Drug expulsion during storage	Storage and administration stability challenges, industrial-scale limitation	^179^
Disadvantages	Low drug loading capacity	High requirement of formulation doses	^180^
	Polydispersity	Undesirable for intravenous administration	^181^
	Agglomeration	Storage issues	^182^
	Storage in refrigerated conditions	Transportation issues, expensive storage costs	^183^
	High operative temperature	Susceptibility of thermolabile drugs	^184^

## Mechanisms of drug release by SLNs/NLCs

 To improve the benefits and avoid the drawbacks, different criteria have been considered for formulation, design and encapsulation rate of nanomedicines.^185^ The encapsulated drug undergoes surface dissolution and degradation of the lipid matrix which results in diffusion of molecules from the matrix into the surrounding tissue.^158^ Drug release from SLNs/NLCs is affected by the localization of the drug,^186^ which can be loaded both in the core matrix and on the surface, the former results in prolonged and sustain release while the latter burst release (quick early-phase release), subsequently conferring a biphasic release profile starting with an quick release owing to the surface-loaded drug continuing by sustained release of more loaded-drug from the lipid matrix.

 The burst release is a feature determined by modifying drug aqueous solubility which is influenced mainly by the surfactant concentration and the temperature via a direct proportion; the higher the last two the higher the burst release. Preparation of SLN/NLC nanoparticles at room temperature has demonstrated no burst release owing to no drug partitioning into water phase and following re-partitioning into lipid phase. Therefore, to decrease the burst release SLNs/NLCs have been prepared without surfactants or surfactants not solubilizing the drug.^185,187^

## *In vivo* fate of SLNs/NLCs after administration

 There are different criteria which determine the *in vivo* fate of nanoparticles: administration route, biological interactions with their environment including distribution, surface adsorption, nanoparticle disaggregation and enzymatic degradation.

 Due to the presence of lipids and waxes in the structure of lipid-based nanoparticles, their fate is highly affected by different pathways and enzymes for their biological interactions, namely as lipases which exist ubiquitously in body and mostly activate by oil/water interface,^188-190^ and actively confer various degradation rates to nanoparticles due to their formulation material ^191-194^ the free fatty acids of degradation have been studied by enzymatic test,^195^ which demonstrated lesser degradation with long-chain fatty acids of triglycerides and surfactants contained in nanoparticles. Surfactants (e.g. poloxamer 407, poloxamer 188) function either to fasten or postpone the degradation process of nanoparticles, as different surfactants (e.g. cetyl palmitate)^192^ have different chain lengths, and this feature could be used in nanoparticle preparation to render a more controlled drug release profile.

 So far, there has been few studies,^196^ approving whether the presence of food in stomach would affect nanoparticles’ in-vivo function or not, and this still remains a dilemma to be solved. In one animal study increased bioavailability and blood half-time was reported with oral administration of lipid nanodispersions,^197^ and in another study the increased absorption of nanoparticles into lymph through intraduodenal route was reported.^198^

## *In vivo* toxicity evaluation of SLNs/NLCs

 Along with their different therapeutic application as DDSs, SLNs and NLCs have been investigated for their in-vivo toxicity/safety profile. Due to their composition of lipids which are physiological components they are generally recognized as safe (GRAS) and better-tolerated nanoparticles than polymeric ones showing lower toxicity,^199,200^ as they are degraded by normal physiological pathways. Nevertheless, the type of lipids and surfactants (emulsifiers) used for their preparation might increase or decrease cell toxicity and even influence encapsulated drug toxicity.^201,202^ Therefore, the toxicity evaluation must include bulk materials, SLNs/NLCs, drug/API itself and drug-encapsulated nanoparticles to analyze thoroughly each component and materials contribution to toxicity. The excipients role for drug encapsulation must be assessed according to the route of administration.^203^

 Different in-vitro tests, among all cell viability (MTT assay) and oxidative stress, have been exploited to assess the cytotoxicity of nanoparticles; the former functions as the color-change of tetrazolium which is the indication of cell death, and the latter demonstrates DNA damages, elevated amounts of reactive oxygen species, lipid peroxidases and alterations in oxidation/reduction glutathione reactions.^204^ MTT assay is the most common method used with different dyes such as Neutral red, Trypan blue,^204-206^ and there are in-vitro and in-vivo experiments on cells proving their low toxicity.^207,208^

## SLNs and NLCs co-delivery strategies

 Through numerous cancer-related studies, it has been proved that broad-spectrum anti-cancer agents will have many benefits in terms of efficacy over monotherapy. SLNs and NLCs could be a promising carrier for co-delivery of anti-cancer, therapeutic nucleic acids and antibiotics,^209^ as they are significantly able to enhance the in-vitro and in-vivo therapeutic efficacy of such drugs. Besides such advantage, co-encapsulation of different drugs in one LPDDS might decrease toxicity of the respective anti-cancer drugs and other adverse effects coming with them separately.^210^

 Another alternative being offered by co-delivery is RNA interference,^211^ especially siRNAs have been exploited for cancer cells to silence the oncogenes expression.^209^ In this context, miRNAs have been investigated and proved to be efficient to regulate genes associated with tumorigenesis.^210^

 In one study,^209^ cationic SLNs for co-delivery of paclitaxel and human myeloid cell leukemia (MCL1) specific siRNA have been investigated and the final result demonstrated enhanced in-vitro and in-vivo efficacy than administering them separately.

 In another similar study,^210^ SLNs were encapsulated for co-delivery of the same active substance with miRNA-34a. The final result was significant in terms of eliminating lung cancer relapse mostly owing to the synergic efficacy and higher inhibition of specific receptors.

 In another study,^212^ the efficacy of Paclitaxel and Verapamil co-loaded SLNs toward breast cancer were investigated to prove the efficiency of verapamil for inhibiting drug efflux transporters (e.g. p-glycoprotein) on multidrug resistance cancer cells. The study demonstrated higher expression downregulation of g-glycoproteins in the specific cancer cells, as well as higher cellular drug uptake and toxicity comparing to Paclitaxel and sole anti-cancer administration.

 In another study on antibiotics,^213^ increased antibacterial activity of Vancomycin was demonstrated. Ion pairing with linoleic acid was exploited for co-delivery and significant effects against Staphylococcus aureus infections which could be interpreted owing to the increased lipophilicity, sustained release of antibiotic and synergistic effect.

 So far different pharmaceutical/biotechnological products have been marketed using lipid-based DDSs (Table 6).

**Table 6:** List of marketed lipid-based oral pharmaceutical products

**Product/Trade name**	**Drug/Molecule**	**Nanotechnology/Dosage form**	**Therapeutic use/Indication**	**Company/Alliance**
Abelcet	Amphotericin B	Nanoliposome/solution	Fungal infections	The Liposome Company Inc
Accutane	Isotretinoin	Emulsion/soft gelatine capsule	Anti-comedogenic	Roche
Agenerases	Amprenavir	Soft gelatine capsule	HIV antiviral	GlaxoSmithKline
ALEC	Dry protein free powder of DPPC-PG	Liposome	Lung diseases in infants	Britannia Pharmaceuticals Ltd
Ambisome	Amphotericin B	Powder	Fungal infections	NeXstar Pharmaceutical Inc
Amphocil	Amphotericin B	Colloidal dispersion	Fungal infections	Sequus Pharmaceutical Inc
Amphotec	Amphotericin B	Nanoliposome/Solution	Fungal infections, leishmaniasis	Sequus Pharmaceutical Inc
Aptivus	tipranavir	Emulsion/soft gelatine capsule	AIDS	Boehringer Ingelheim
Atragen	Tretinoin	Liposome	Acute myeloid leukemia	Aronex Pharmaceuticals Inc
Avodart	Dutasteride	Emulsion	Benign Prostatic Hyperplasia	GSK
Avian retrovirus vaccine	Killed avian retrovirus	Suspension	Chickenpox	Vineland Laboratories
Cipro	Ciprofloxacin	Oral suspension	Antibiotic	Bayer
Convulex	Valproic acid	Soft gelatine capsule	Antiepileptic	Pharmacia
DaunoXome	Daunorubicin citrate	Solution	Kaposi sarcoma in AIDS	NeXstar Pharmaceutical Inc/ Galen Ltd
Depakene	Valproic acid	Emulsion	Epilepsy	Abbott
Depocyt	Cytarabin	Nanoliposome/Solution	Lymphomatous meningitis	Pacira Pharmaceuticals Inc
DepoDur	Morphine	Suspension	Post-surgical pain reliever	Pacira Pharmaceuticals Inc
Doxil	Doxorubicin	Solution	Metastatic ovarian, Kaposi sarcoma in AIDS	Sequus Pharmaceutical Inc
Emend	Aprepitant	Nanosuspensions/Capsule	Antiemetic	Merck-Elan Drug Delivery
Epaxal Berna Vaccine	Inactivated hepatitis-A virions	Suspension	Hepatitis A	Swiss serum & vaccine institute
Estrasorb	estradiol	Topical emulsion	Menopausal therapy	Novavax
Evacet	Doxorubicin	Liposome	Metastatic breast cancer	The liposome company
Fenogal	Fenofibrate	Tablet	Anti hyperlipproteinomic	Genus
Fortovase	saquinavir	Spontaneously emulsifying systems/soft gelatine capsule	HIV antiviral	Roche
Fungizone	Amphotericin B	Solution	Fungal infections	Bristol-Myers Squibb
Gengraf	Cyclosporin A/III	Spontaneously emulsifying systems/hard gelatine capsule	Immuno-suppressant	Abott
Hectoral	Doxercalciferol	Emulsion	Calcium regulator	Bone care
Juvela	Tocopherol nicotinate	Capsule	Hypertension, hyperlipidemic	Eisai Co.
Kaletra	Lopinavir & Ritonavir	Emulsion/oral solution	HIV antiviral	Abott
Lamprene	Clofazamine	Emulsion	Leprosy	Alliance laboratories/ Geigy
Lipirex	fenofibrate	Hard gelatine capsule	hyperlipidemia or mixed dyslipidemia	Sanofi-Aventis
Marinol	Dronabionol	Emulsion	Anoxeria	Roxane
Megace ES	Megestrol acetate	Nanosuspension	anorexia, cachexia, weight loss in HIV patients	Par Pharmaceuticals- Elan Drug Delivery
MiKasome	Amikacin	Liposome	Bacterial infection	NeXstar Pharmaceutical Inc
Neoral	Cyclosporin A/I	Emulsion	Immunosuppressant	Novartis
Norvir	Ritonavir	Spontaneously emulsifying systems/soft gelatine capsule	HIV antiviral	Abott
Nyotran	Nystatin	Liposome	Fungal infections	Aronex pharmaceuticals Inc
Panzem NCD	2-Methoxy estradiol	Nanosuspension	anti-proliferative and anti-angiogenic effect	EntreMed Inc.
Prometrium	Progesterone	Emulsion	Endometrial hyperplasia	Solvay
Rapamune	Sirolimus	Nanosuspensions/Tablet	Immunosuppressant	Wyeth Pharmaceuticals – Elan Drug Delivery
Restandol	Testosterone undecanoate	Capsules	Hormone replacement therapy	Organon laboratories
Rocaltrol	Calcitriol	Emulsion/soft gelatine capsule	Calcium regulator	Roche
Sandimmune Neoral	cyclosporine A/I	Spontaneously emulsifying systems/soft gelatine capsule	Immunosuppressant	Novartis
Sustiva	Efavirenz	Capsules	HIV antiviral	Bristol-Meyers
Targretin	bexarotene	Soft gelatine capsule	liver cancer	Novartis
Topex-Br	Terbutalinesulphate	Syrup	Asthma	Ozone Pharmaceuticals Ltd
Tricor	Fenofibrate	Nanosuspensions/Tablet	Antihyperlipidemic agent	Abbott Laboratories
Triglide	Fenofibrate	Nanosuspensions/Tablet	Antihyperlipidemic agent	Skye Pharma-First Horizon
Ventus	Prostaglandin-E1	Liposome	Systemic inflammatory disease	The Liposome Company
Vesanoid	tretinoine	Emulsion/soft gelatine capsule	Acne	Roche
VincaXome	Vincristine	Liposome	Solid tumors	NeXstar Pharmaceutical Inc
Zemplar	Paricalcitol	Emulsion	Calcium regulator	Abbott

## LBDDSs formulations to enhance oral delivery of hydrophobic peptide and protein therapeutics

 Although there have been promising achievements with LBDDSs for oral delivery of hydrophobic peptide and protein therapeutics, the hydrophilic peptide and protein delivery still remains a challenge and limited to the in-vitro and in-vivo experiments with no product in the pharmaceutical market.

 There have been many studies using these lipid-based scaffolds to prove their potential to be exploited in future studies. there have been numerous studies of Insulin as a hydrophilic peptide encapsulated in micelles, microemulsion NPs and nanocapsules with in-situ and in-vivo experiments on rat with promising results as enhanced permeability, bioavailability and efficacy.^214-220^ In one study SK&F 106760 (a hydrophilic RGD peptide) in the form of microemulsion exploited in in-vivo experiments and demonstrated 50-fold elevated bioavailability.^221^ In another study Vasopressin was encapsulated as microemulsion in in-situ experiments and resulted in enhanced bioavailability.^222^ In one study EGF (a single-chain polypeptide) was encapsulated in microemulsions for in-vivo experiments of gastric ulcer in rats and showed increase efficacy.^223^ In one study on ß-lactamase in-vivo experiments resulted in enhanced 2.5-fold bioavailability.^224^ N-acetylglucosaminyl and N-acetylmuramyl dipeptide were exploited in one study which demonstrated 10-fold increased bioavailability.^225^ In another study Leuprolide acetate was encapsulated in microemulsion for in-vivo experiments and proved increased efficacy.^226^ Two experiments of lipid mixtures with Hexarelin and DMP 728 (Cyclic peptide fibrinogen antagonist) as encapsulated drugs with in-situ experiments showed 20-fold and 3-fold intestinal permeability and bioavailability, respectively.^227,228^ In the latter study in dog, DuP 532 (an Angiotensin II antagonist) was encapsulated in microemulsion in in-vivo experiments which resulted in 3-fold bioavailability.^228^ In three studies in rat and pig, calcitonin was encapsulated in mixed micelles and emulsion for in-situ and in-vivo experiments which demonstrated increased permeability and efficacy and 4-fold hypocalcemia response.^229-231^ Human growth hormone was encapsulated in in-vivo studies on rabbit and showed 3.3% increased bioavailability.^232^

## Regulatory status, commercialization plan and safety information

 The status of excipients should be assessed with the regulatory authorities before any pharmaceutical product’s introduction into the market ^233^ but the expenses of in-vivo toxicity studies are prohibitive for the companies. Such a challenge is happening mainly with the polymeric NPs as there are few of them in the market but lipid NPs owing to their various applications of oils, fats, stabilizer and surfactants have introduced oral and dermal products. The majority of the introduced excipients so far for lipid NPs synthesis are biodegradable, biocompatible and are approved as safe, but some are toxic at high concentrations.^234^ In this context the FDA has published guide lists of safe materials and substances (GRAS) and inactive ingredient guide (IIG) for excipients that are approved for exploiting in the pharmaceutical products in the market.^235^ These lists explain and provide insights regarding the appropriate excipient concentration for each administration route and the approved inactive ingredients used for a specific route can be used in all the new formulations. This facilitates the process of synthesizing new formulation as the necessary information can be extracted from the GRAS and IIG. Therefore, such excipients are assessed as substances of a drug not individually as from a “scientific point of view” excipients are a major part of the drug formulation.

 Nevertheless, there are other challenges to consider from a “regulatory point of view”; preclinical and clinical studies addressing safety issues and in-vivo manifestations of the LBDDSs in terms of clinical therapeutic efficacy. In-vivo immunological and stability findings toward oils and lipid excipients must be reported to provide in-depth information for the regulatory authorities.^236^

 Besides, factors coming from the biopharmaceuticals are required to be evaluated toward the drug or excipients and this might have paradoxical in-vitro results with in-vivo results due to the physiology of GI tract. Various experiments must be designed and conducted to characterize and recognize the interactions happening among excipients, in-vivo physiological conditions and the drug.^237^

 In order to understand and characterize the in-vivo fate of drugs encapsulated in LBDDSs, a consortium of academic and industrial scientists has been established (http://www.lfcsconsortium.org) which designs experiments to evaluate the function of LBDDSs dispersion and digestion as vital criteria.

## Conclusion

 Nanotechnology offers promising strategies for enhancing oral bioavailability and therapeutic efficacy of a vast range of drugs; conventional chemical drugs with poor water solubility and biotechnological, peptide and protein therapeutics and biopharmaceuticals. The unique physicochemical features of peptide and protein therapeutics pose challenge for their oral delivery. Hence, their success in site delivery highly depends on technologies and methods to modify these two features not influencing their biological function. In the recent decades numerous DDSs have been introduced and offered by nanotechnology to achieve as high successful delivery as possible and LBDDSs among all has been under investigation owing to their potential for oral delivery of hydrophilic, hydrophobic and lipophilic peptide and protein therapeutics.

 LBDDSs enhance solubility and bioavailability of drugs offering strategies such as gastrointestinal lymphatic transport, altering physiological and biochemical properties of gastrointestinal barriers, elevated solubilization and prolonged gastrointestinal retention. Although, such improvements rely on the encapsulation/loading rate and intrinsic composition of the material used during the fabrication process. Obviously, the choice of materials, such as excipients, will influence the success of delivery route which is determined both by lipid formulation design and peptide/protein molecule emphasizing that each peptide/protein-loaded LBDDS must be designed uniquely. Such material must be in correlation with the drug of choice to achieve the maximum therapeutic efficacy and in-site dose.

 Most of the scaffolds described in this review article suggest promising alternatives to overcome gastrointestinal enzymatic degradation and poor membrane penetration. Further systematic studies are required to evaluate their in-vivo efficacy in terms of peptide-/protein-based oral drug delivery. Besides “pharmaco-biotechnological” challenges mentioned in this review such as membrane permeability, protease stability, delivery strategies and increased circulation half-life, there are inevitably several “industrial” challenges as well which finally hamper their industrial scale production and consequently their biomedical translation from lab to pharmaceutical market. “Oral bioavailability” still remains the main challenge of peptide/protein-based drug delivery. These factors could be addressed as materials cost, drug potential market feedback, regulatory status, simple industrial-scale fabrication, financial schemes for required instruments, patient compliance administration and high adaptability to human diverse pharmacokinetics.

## Competing Interests

 The author declares no conflict of interest.

## Ethical Approval

 There is none to be disclosed.

## Funding

 The author declares no funding/support.
